# The Fabrication and High-Efficiency Electromagnetic Wave Absorption Performance of CoFe/C Core–Shell Structured Nanocomposites

**DOI:** 10.1186/s11671-018-2474-9

**Published:** 2018-03-01

**Authors:** Gengping Wan, Yongming Luo, Lihong Wu, Guizhen Wang

**Affiliations:** 1 Faculty of Environmental Science and Engineering, Kunming University of Science and Technology, Kunming, 650500 China; 2 Key Laboratory of Tropical Biological Resources of Ministry of Education, Hainan University, Haikou, 570228 China

**Keywords:** Electromagnetic wave absorption, Core–shell, Carbon, CoFe

## Abstract

CoFe/C core–shell structured nanocomposites (CoFe@C) have been fabricated through the thermal decomposition of acetylene with CoFe_2_O_4_ as precursor. The as-prepared CoFe@C was characterized by X-ray powder diffraction, X-ray photoelectron spectroscopy, Raman spectroscopy, transmission electron microscopy, and thermogravimetric analysis. The results demonstrate that the carbon shell in CoFe@C has a poor crystallization with a thickness about 5–30 nm and a content approximately 48.5 wt.%. Due to a good combination between intrinsic magnetic properties and high-electrical conductivity, the CoFe@C exhibits not only excellent absorption intensity but also wide frequency bandwidth. The minimum RL value of CoFe@C can reach − 44 dB at a thickness of 4.0 mm, and RL values below − 10 dB is up to 4.3 GHz at a thickness of 2.5 mm. The present CoFe@C may be a potential candidate for microwave absorption application.

**Electronic supplementary material:**

The online version of this article (10.1186/s11671-018-2474-9) contains supplementary material, which is available to authorized users.

## Background

Developing novel microwave absorption materials (MAMs) is considered as one of the effective methods to solve the increasingly serious electromagnetic (EM) interference problems since MAMs can absorb unwanted EM energies by converting them into other energy types [1–5]. Up to now, a variety of MAMs have been exploited in order to meet with the requirements of wide bandwidth, strong absorption, low density, and good stability [6–9]. Research confirmed that nanostructured core–shell absorbers could combine multiple wave loss mechanisms and achieve high-efficiency wave absorption performance [10–14]. For example, Cao et al. reported that 3D Fe_3_O_4_ nanocrystals decorating on carbon nanotubes exhibited the minimum RL value of − 52.8 dB at 12.8 GHz [15]. Wang et al. synthesized flower-like ZnO coated by Ni nanoparticles via an atomic layer deposition-assisted strategy [16]. Ni-coated ZnO nanohybrids showed superior EM wave absorbing characteristics compared with pure ZnO. Du et al. reported the synthesis of Fe_3_O_4_@C core–shell composites through in situ polymerization of phenolic resin and subsequent high-temperature carbonization [17]. Their results revealed that the microwave absorption properties of the Fe_3_O_4_@C were greatly enhanced. Wu et al. fabricated the elliptical Fe_3_O_4_/C core–shell nanorings via a one-pot hydrothermal route, and the composites showed an enhanced low-frequency microwave absorption [18].

Metallic magnetic materials are a kind of potential microwave absorbers and have attracted much attention due to their large saturation magnetization and high Snoek limit at high frequencies [19, 20]. For example, the 3D nets constructed by dispersing nickel chains showed excellent microwave absorption capacity even at relatively high temperature of 373 K [21]. Dual dielectric resonance and two strong absorption peaks were achieved by the cobalt nanochains [22]. Nevertheless, single-component metallic magnetic materials usually show an unsatisfactory high-frequency permeability due to eddy-current effect, which hampers their further applications [12, 23, 24]. Recently, much effort has been put to address the problem [25–28]. Thereinto, synthesizing metallic magnetic particles in a nano scale and encapsulating them with a thin dielectric layer to isolate from each other could efficiently inhibit eddy-current effect and improve their microwave absorption performance. As a prominent representative of dielectric absorbing material, carbon materials have outstanding properties that make them superior candidates as ideal shell materials, namely, excellent electrical conductivity and good stability [29–31]. Zhang et al. synthesized FeCo@C nanoflakes and found that the orientation could decrease the absorber thickness and increase the absorption performance [32]. Zeng et al. reported that CoFe@C core–shell nanocomposites synthesized by a template-engaged approach exhibited microwave absorption performance with an effective absorption bandwidth of 4.3 GHz [33]. However, developing a facile method to fabricate metallic magnetic materials/carbon composites with a well-defined core–shell structure including the high yield and uniformity remains a big challenge.

In this work, we demonstrated a facile and efficient method for the preparation of uniform CoFe/C core–shell structured nanocomposites (CoFe@C) and investigated its microwave absorption properties. The as-prepared CoFe@C shows excellent microwave absorption performance and is very promising as a strong absorption and wide bandwidth microwave absorber.

## Methods/Experimental

### Synthesis of CoFe_2_O_4_

CoFe_2_O_4_ samples were synthesized via a facile method. The typical synthesis process of CoFe_2_O_4_ is as follows: 2.5 g of CoCl_2_·6H_2_O and 5.6 g of FeSO_4_·7H_2_O were dissolved in 80 mL of deionized water and then transferred into oil bath heating at 80 °C, under vigorous stirring for 1 h. Subsequently, 30 mL 1 M oxalic acid solution was heated to boiling with magnetic stirring and added into the above solution slowly under constant stirring to form a final black precipitation, and then cooled by ice-water mixture. The black precipitates were collected by centrifugation and further washed several times with water and ethanol, respectively, and then dried at 60 °C under vacuum for 12 h. Subsequently, the precipitates were transferred to a muffle furnace and heated at 600 °C for 1 h. The temperature was raised at a heating rate of 1 °C min^− 1^.

### Synthesis of CoFe@C

The as-obtained CoFe_2_O_4_ were loaded into a porcelain boat, transferred to a tube furnace, and put at the center of the furnace. After evacuation, a stream of acetylene (an atmosphere pressure) was introduced. The reaction was performed at 400 °C for 1 h (5 °C min^− 1^) at atmospheric pressure. After the apparatus was cooled to room temperature, the CoFe@C was obtained.

### Morphology, Structure, and Magnetic Properties’ Characterization

Transmission electron microscopy (TEM) and high-resolution TEM (HRTEM) images were taken on a JEOL JEM-2100 microscope instrument. The crystal structure was examined using X-ray diffraction (XRD) with Cu Kα radiation on a Bruker D8 Advance diffractometer. X-ray photoelectron spectroscopy (XPS) was acquired using an AXIS SUPRA spectrometer with a monochromatic Al Kα (1486.6 eV) source. Thermogravimetric (TG) results were obtained by a thermal analysis system (Q600, TA, USA) using a heating rate of 10 °C min^− 1^ in air. Raman spectroscopy was performed on a Renishaw inVia Reflex Raman microscope using 532 nm green laser excitation. The magnetic properties were measured using a MicroMag 2900/3900 alternating gradient magnetometer.

### Microwave Absorption Properties

The specimens for measuring the microwave absorption properties were prepared by uniformly mixing 50 wt.% of CoFe_2_O_4_ or CoFe@C with paraffin and pressing the mixture into a cylindrical shape. Then the cylinder was cut into a toroid of 7.00 mm outer diameter and 3.04 mm inner diameter for measurement. The relative permeability and permittivity values of the mixture were determined and obtained by measuring *S*_11_ and *S*_21_ parameters at 2–18 GHz with a vector network analyzer (Agilent N5230A) by using the transmission/reflection coaxial line method.

## Results and Discussion

**Fig. 1:**
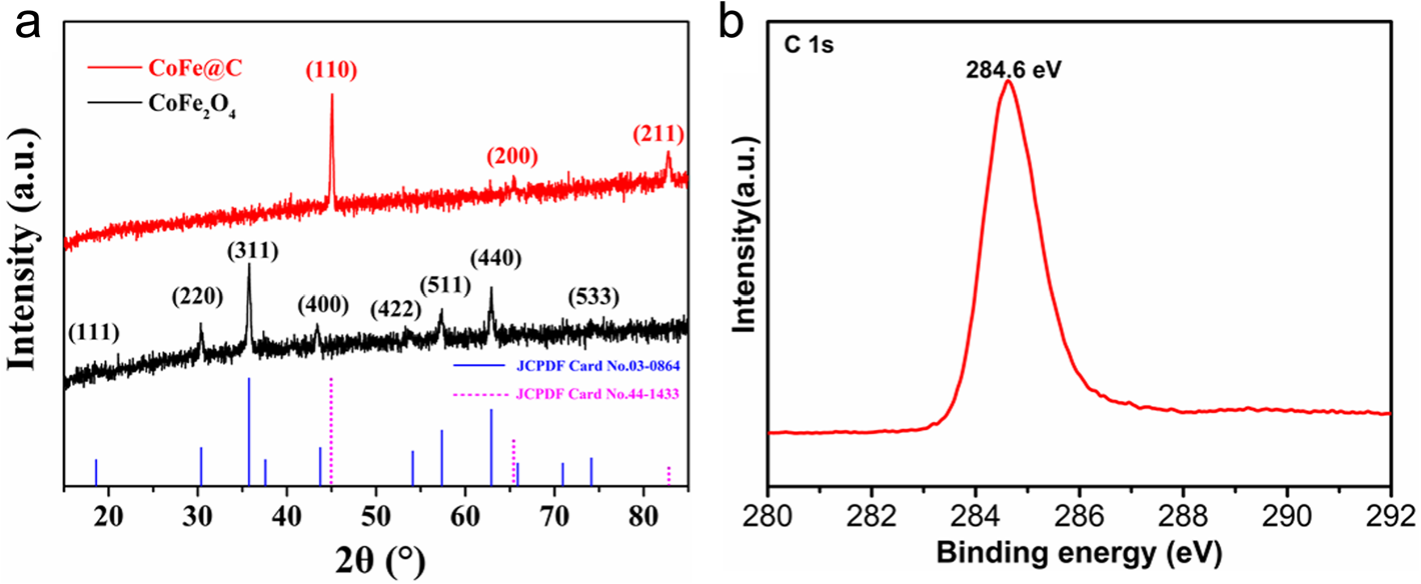
**a** XRD patterns of CoFe_2_O_4_ and CoFe@C. **b** C 1s XPS spectrum of CoFe@C

We performed XRD analysis to investigate the crystal structure and purity of the samples. Figure 1a displays the XRD patterns of CoFe_2_O_4_ and CoFe@C. For CoFe_2_O_4_, all characteristic peaks of sample match very well with the inverse spinel structure with the lattice parameters of *a* = 8.377 Å and *c* = 8.377 Å, which is consistent with the reported data (JCPDS File No. 03-0864). For CoFe@C, three obvious typical peaks can be indexed as the (110), (200), and (211) reflections of Fe-Co alloy phase (JCPDS no. 44-1483). No obvious characteristic peaks from crystalline graphite are detected, indicating that the coated carbon shell is amorphous [34]. Furthermore, the typical XPS survey spectra of CoFe@C shows the presence of C, O, Fe, and Co (Additional file 1: Figure S1). XPS is a surface analysis tool to investigate the superficial composition and chemical state of elements on the surface of sample. It should be noted that the intensity of Fe 2p and Co 2p peaks for CoFe@C is very weak owing to the thick carbon coating on the surface of CoFe. For C 1s, a characteristic peak at 284.5 eV (Fig. 1b) corresponding to the sp^2^-hybridisation state of graphite carbon can be observed [35]. XPS results confirm that a uniform carbon layer was successfully coated on the surface of CoFe. CoFe@C core–shell composites would lead to improved dielectric properties due to good electrical conductivity and enhanced interfacial polarizations, which is beneficial to their good wave absorbing properties.

**Fig. 2:**
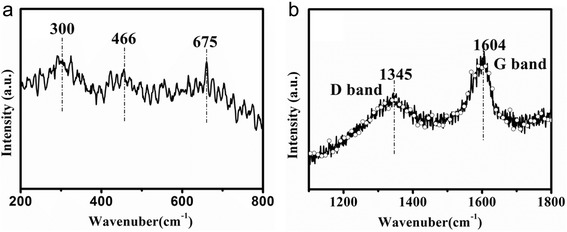
Raman spectra of **a** CoFe_2_O_4_ and **b** CoFe@C

Raman spectroscopy can be used to study the information on the coordination of metal ions. Figure 2a shows the Raman spectrum of CoFe_2_O_4_. CoFe_2_O_4_ has the cubic inverse spinel structure similar to Fe_3_O_4_ attributed to the space group ${\mathrm{O}}_{\mathrm{h}}^7\left(\mathrm{Fd}\overline{3}\mathrm{m}\right)$ [36]. The low-frequency vibrations (below 600 cm^− 1^) are assigned to the motion of oxygen around the octahedral lattice site whereas the higher frequencies can be attributed to oxygen around tetrahedral sites [37]. In this work, the mode at 682 cm^− 1^ is characteristic of the tetrahedral site. The bands at 470 and 300 cm^− 1^ correspond to Co^2+^ at octahedral sites [38]. We also investigated the presence of carbon in the CoFe@C samples by Raman spectroscopy. Figure 2b is the Raman spectrum of CoFe@C in the range of 1100–1800 cm^− 1^. The peak located at 1345 cm^− 1^ corresponds to the presence of sp^3^ defects of carbon (D-band). The peak at 1604 cm^− 1^ represents the characteristic for graphitic sheets (G-band). In this work, the G-band peak of the CoFe@C shifts to a higher wavelength number in comparison with that of well crystalline graphite structures (1575 cm^− 1^), suggesting that the carbon shell is of highly disorderly [39–41].

**Fig. 3:**
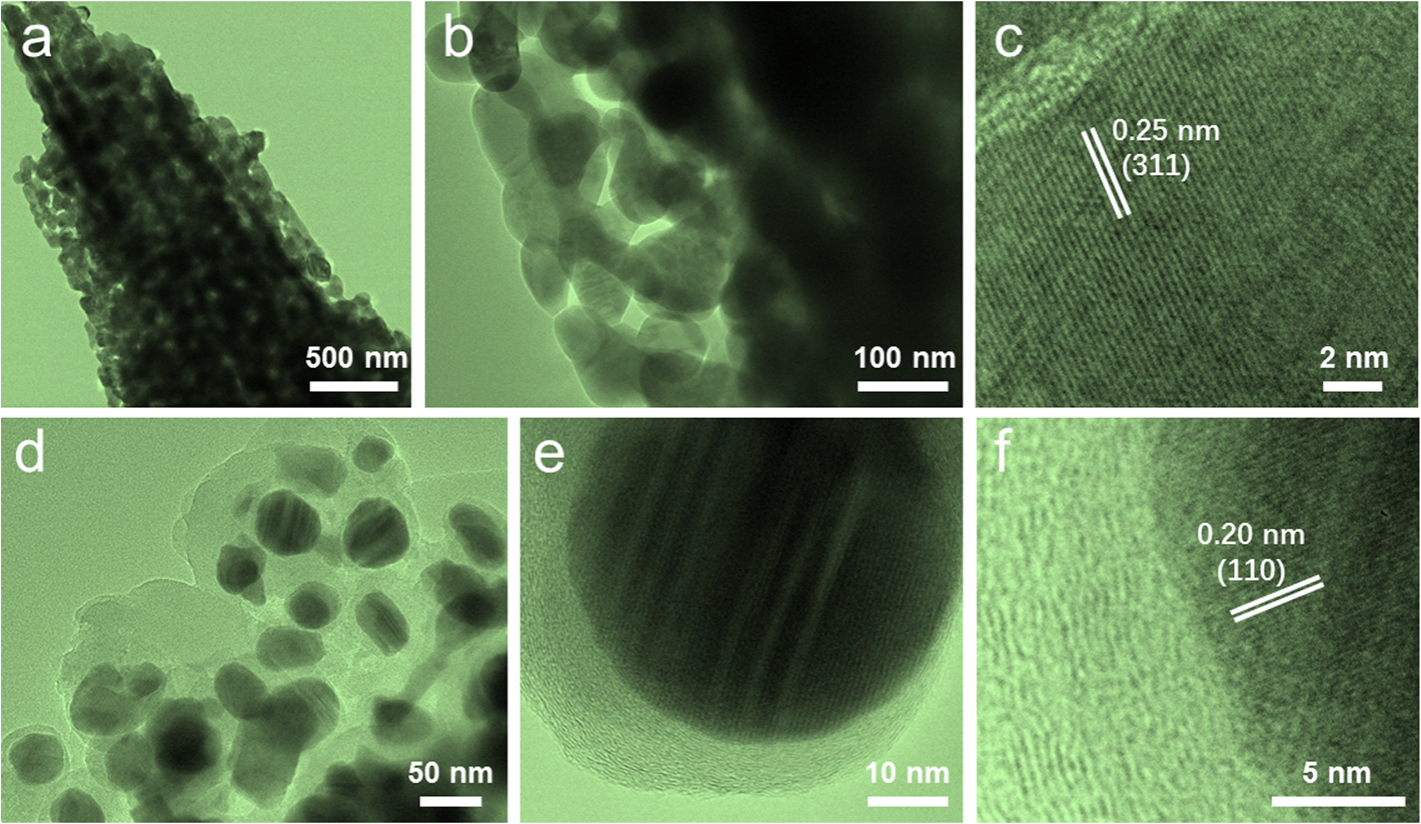
**a**, **b** TEM and **c** HRTEM images of CoFe_2_O_4_. **d**, **e** TEM of CoFe@C and **f** HRTEM images of CoFe@C

TEM characterization on CoFe_2_O_4_ and CoFe@C was performed to investigate the microstructure and morphology. Figure 3a, b exhibits that the CoFe_2_O_4_ has a mesoporous structure. All these pores are located among adjacent particles produced from a great quantity of gases release of oxalate precursors during thermal decomposition. The HRTEM image displays the legible lattice fringes of 0.25 nm corresponding to (311) plane of inverse spinel structured CoFe_2_O_4_, indicating the highly crystalline nature of the mesoporous particles (Fig. 3c). Through a simple heating process in acetylene, the CoFe@C nanoparticles could be obtained. As shown in Fig. 3d, e, the average size of CoFe nanoparticles is in the range of 40–70 nm in diameter. The carbon shell has a poor crystallization with a disorderly graphitized carbon layer of about 5–30 nm in thickness. The clear crystalline lattice spacing of 0.20 nm from the dark core part can be indexed as (110) crystal planes of CoFe alloy (Fig. 3f). The interplanar distances of the surface carbon layers are around 0.34 nm, which is in agreement with the previous reports.

**Fig. 4:**
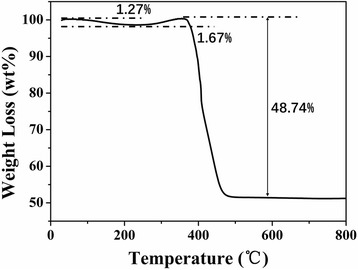
TG curves of CoFe@C

We performed TGA to evaluate the carbon content in CoFe@C. Figure 4 shows the TG curve of CoFe@C. It is found that the weight loss is about 1.27% for CoFe@C from room temperature to 200 °C, which is ascribed to the loss of surface adsorbed water and other adsorbed organic functional groups. From 200 to 380 °C, a weight increase around 1.67% should be from the oxidation weight gain of CoFe. Next, an obvious weight loss is found resulting from the thermal decomposition of carbon. The TG curve keeps stable after 485 °C and the total weight loss is about 48.74%. On the basis of these results, the carbon content in CoFe@C is evaluated to be approximately 48.5 wt.%.

**Fig. 5:**
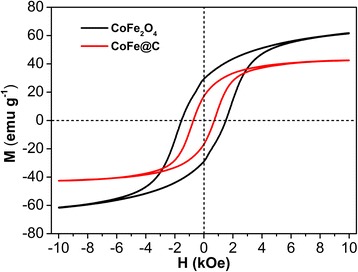
The hysteresis loops of the CoFe_2_O_4_ and CoFe@C at room temperature

The magnetic hysteresis loops of CoFe_2_O_4_ and CoFe@C were measured at room temperature. As shown in Fig. 5, the values of magnetic saturation (*M*_*s*_) and the coercivity (*H*_ci_) for CoFe_2_O_4_ are 61.7 emu g^− 1^ and 1536.8 Oe, respectively. According to a previous study, the samples with bigger grain sizes possess higher value of *M*_*s*_ [42]. In this work, the relatively high *M*_*s*_ value for CoFe_2_O_4_ compared with several literatures should be attributed to the big crystalline grain size as confirmed from TEM images [43–45]. For CoFe@C, the *M*_*s*_ value is 42.6 emu g^− 1^ and the *H*_ci_ is 729.2 Oe. The coercivity is larger while the value of saturation magnetization is smaller than that of bulk FeCo alloys [46]. However, it is comparable with other reported CoFe or CoFe@C composites [32, 33, 47]. Some slight differences may be ascribed to the Co/Fe proportion, coating of carbon layer and grain size. The good intrinsic magnetic properties for the CoFe@C would contribute to the high magnetic loss, which is favorable for enhancing their microwave absorption performance.

**Fig. 6:**
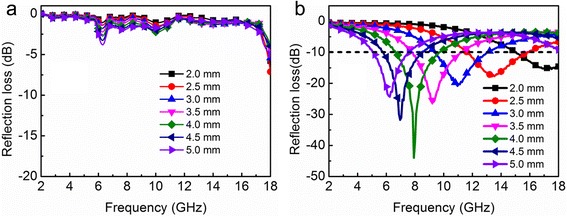
Reflection loss curves of **a** CoFe_2_O_4_ and **b** CoFe@C at different thicknesses

The microwave absorption properties of CoFe_2_O_4_ and CoFe@C were investigated by mixing 50 wt.% of the samples with paraffin. Figure 6 shows the typical relationship between reflection loss (RL) and frequency at different thickness. It is observed from Fig. 6a that CoFe_2_O_4_ exhibits poor wave absorption performance with a minimum RL value of − 7.1 dB at the thickness of 2.5 mm. Moreover, the minimum RL value and absorption peaks show no obvious change with the variation of sample thickness. The microwave absorption properties of CoFe@C involved intensity and bandwidth exhibit significant enhancement (Fig. 6b). To be specific, the minimum RL values of CoFe@C with thicknesses of 2.0, 2.5, 3.0, 3.5, 4.0, 4.5 and 5 mm are − 15.5 dB (at 17.1 GHz), − 17.9 dB (at 13.3 GHz), − 20.8 dB (at 10.9 GHz), − 26.1 dB (at 9.3 GHz), − 44.0 dB (at 7.9 GHz), − 31.8 dB (at 7.0 GHz), and − 24.4 dB (at 6.2 GHz), respectively. The RL values less than − 10 dB for CoFe@C (thickness of 2.5 mm) are in the ranges of 11.6–15.9 GHz corresponding to a bandwidth of 4.3 GHz. In general, materials with RL values below − 10 dB (90% absorption) are regarded as suitable EM wave absorbers. Therefore, the present CoFe@C may be a potential candidate for microwave absorption application.

**Fig. 7:**
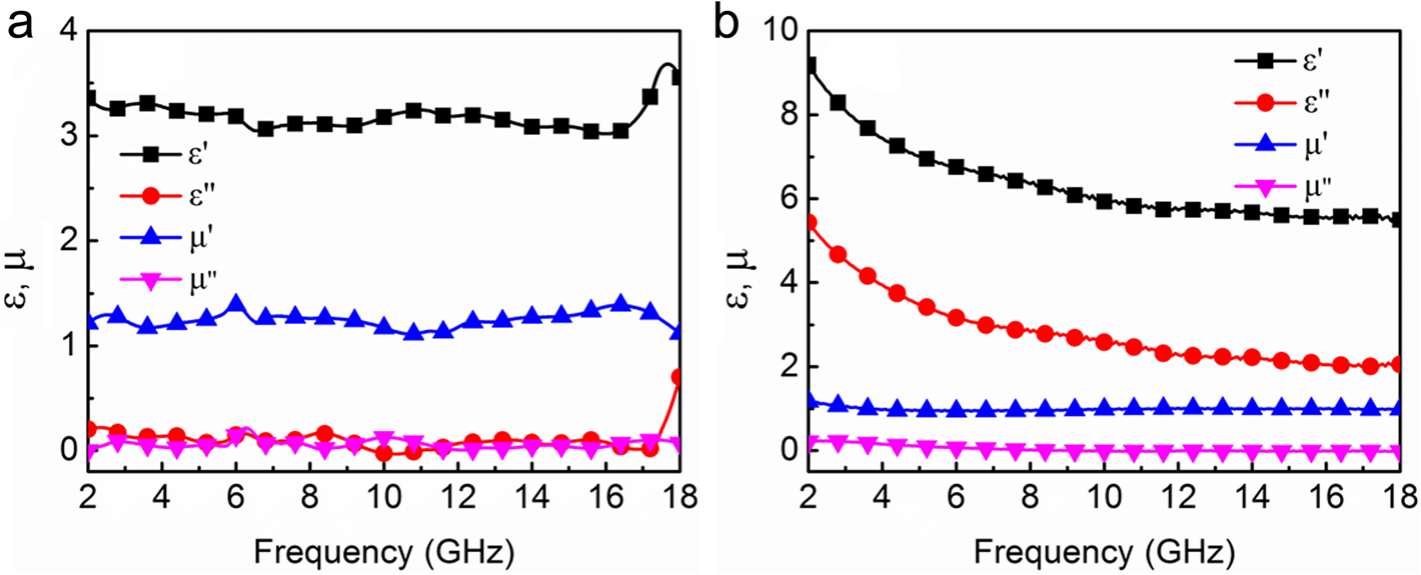
Frequency dependence of real and imaginary parts of complex permittivity and permeability of **a** CoFe_2_O_4_ and **b** CoFe@C

To reveal the possible EM wave absorption mechanism, the complex permittivity (*ε*_r_ = *ε*′ – *jε*″) and complex permeability (*μ*_r_ = *μ*′ − *jμ*″) of the CoFe_2_O_4_ and CoFe@C are given in Fig. 7. It is well known that the real and imaginary parts of complex permittivity and permeability represent electric and magnetic energy storage and dissipation capability, respectively. As shown in Fig. 7a, the *ε*′ and *μ*′ values for CoFe_2_O_4_ remain nearly unchanged and are in the range of 3.1–3.8 and 1.1–1.4, respectively. Meanwhile, CoFe_2_O_4_ has a very small *ε*″ (0.1–0.5) and *μ*″ (0–0.11) values. These results indicate that both dielectric and magnetic loss for CoFe_2_O_4_ is low, which should be responsible for the poor microwave absorption performance. For CoFe@C, it can be seen in Fig. 7b that the values of complex permittivity are obviously higher than those of the CoFe_2_O_4_ in the whole frequency range. With the increasing frequency, the *ε*′ and *ε*″ values show a slow decline and are in the range of 5.5–9.1 and 2.0–5.4, respectively. The *μ*′ values of CoFe@C are in the range of 0.98–1.2, whereas the *μ*″ values are in the range of 0–0.23, suggesting that CoFe@C has a larger magnetic loss in the microwave frequency range than that of CoFe_2_O_4_.

In this work, the high *ε*′ and *ε*″ values should be ascribed to the good conductivity of CoFe@C composites. According to Cao’s electron-hopping model, the well-conductivity CoFe@C allows electrons to migrate and hop, and thus can greatly consume the electromagnetic energy, leading to enhanced dielectric loss [48–51]. Moreover, two peaks at *f* = ~ 5.2 and ~ 11.1 GHz (Additional file 1: Figure S2) from the Cole-Cole plots indicate the existence of dual relaxation behaviors in CoFe@C samples. These relaxations are probably derived from the surface functions, defects, and interfacial polarizations in CoFe@C composites. In addition, the magnetic loss also contributes to the electromagnetic wave attenuation of CoFe@C composites. Eddy current effects, natural resonance, and exchange resonance are three key wave loss sources in the microwave region. As shown in Additional file 1: Figure S3, the *μ*″(*μ*′)^−2^*f*^− 1^ for CoFe@C is not a constant value, suggesting eddy current is not the dominating magnetic loss mechanism. Instead, two peaks at ~ 3 and 12.5 GHz can be found and are indexed to natural resonance and exchange resonance. We also calculated the dielectric loss tangents (tan *δ*_E_ = *ε*″/*ε*′) and magnetic loss tangents (tan *δ*_M_ = *μ*″/*μ*′) of CoFe@C and CoFe_2_O_4_, in which the maximum values of tan *δ*_E_ and tan *δ*_M_ are 0.706 and 1.370, respectively (Additional file 1: Figure S4). The relatively high values of tan *δ*_E_ and tan *δ*_M_ further reveal that CoFe@C possesses intense dielectric and magnetic loss.

**Fig. 8:**
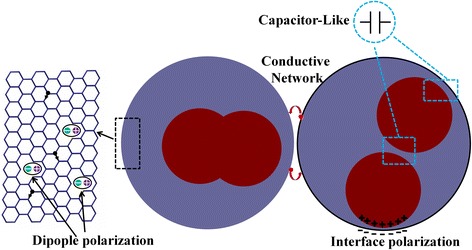
Schematic illustration for the microwave absorption mechanism of CoFe@C

Therefore, the excellent microwave absorption performance for CoFe@C should be attributed to the appropriate combination of dielectric–magnetic multiple loss mechanisms. As shown in Fig. 8, the electrons could easily migrate and hop between two well conductive CoFe@C and form micro-current networks, resulting in eminent conduction loss. This is consistent with Cao’s model [52, 53]. The dipole polarization originated from the functional groups, defects, and interface between carbon layers and CoFe nanoparticles is another important loss mechanism. Cao et al. proposed that the capacitor-like structures at the interfaces could also effectively attenuate the power of incident EM waves [54]. Magnetic loss for CoFe@C mainly derives from natural resonance and exchange resonance due to an effective inhibition of eddy-current effect by the uniform carbon layers.

## Conclusions

In summary, we develop a novel method to fabricate the CoFe/C core–shell structured nanocomposites (CoFe@C) for microwave absorption application. The as-prepared CoFe@C manifest remarkable microwave absorption properties including strong absorption and wide bandwidth. The RL values below − 10 dB cover the frequency range of 11.6–15.9 GHz (2.5 mm). The minimum RL value can reach − 44.0 dB when the match thickness is 4.0 mm. The excellent microwave absorption properties are ascribed to the effective combination of dielectric–magnetic multiple loss mechanisms.

## Additional file

Additional file 1: **Figure S1.** XPS survey spectra of CoFe@C. Figure S2. Cole-Cole plots of CoFe@C. Figure S3. The plot of *μ*″(*μ*′)^−2^*f*^− 1^ vs. frequency for the CoFe@C. Figure S4. Frequency dependence of dielectric loss tangents and magnetic loss tangents of the (A) CoFe_2_O_4_ and (B) CoFe@C. (DOCX 580 kb)

## Abbreviations

CoFe@C: CoFe/C core–shell structured nanocomposites
EM: Electromagnetic
HRTEM: High-resolution TEM
MAMs: Microwave absorption materials
TEM: Transmission electron microscopy
TG: Thermogravimetric
XPS: X-ray photoelectron spectroscopy
XRD: X-ray diffraction
