# Supplementary material for: The Fabrication and High-Efficiency Electromagnetic Wave Absorption Performance of CoFe/C Core–Shell Structured Nanocomposites
Source: Nanoscale Res Lett. 2018 Mar 1;13:68. doi: 10.1186/s11671-018-2474-9 (PMC5834946; doi:10.1186/s11671-018-2474-9)
Supplement: Supplementary file 1 — Figure S1. XPS survey spectra of CoFe@C. Figure S2. Cole-Cole plots of CoFe@C. Figure S3. The plot of μ″(μ′)−2f− 1 vs. frequency for the CoFe@C. Figure S4. Frequency dependence of dielectric loss tangents and magnetic loss tangents of the (A) CoFe2O4 and (B) CoFe@C. (DOCX 580 kb) [file 11671_2018_2474_MOESM1_ESM.docx]

**Additional file 1**

The fabrication and high-efficiency electromagnetic wave absorption performance of CoFe/C core-shell structured nanocomposites

Gengping Wan^1,2^, Yongming Luo^1^*, Lihong Wu^2^ and Guizhen Wang^2^*

*Correspondence: environcatalysis222@yahoo.com; wangguizhen0@hotmail.com

^1^Faculty of Environmental Science and Engineering, Kunming University of Science and Technology, Kunming 650500, China

^2^Key Laboratory of Tropical Biological Resources of Ministry of Education, Hainan University, Haikou 570228, China





**Figure S1** XPS survey spectra of CoFe@C.


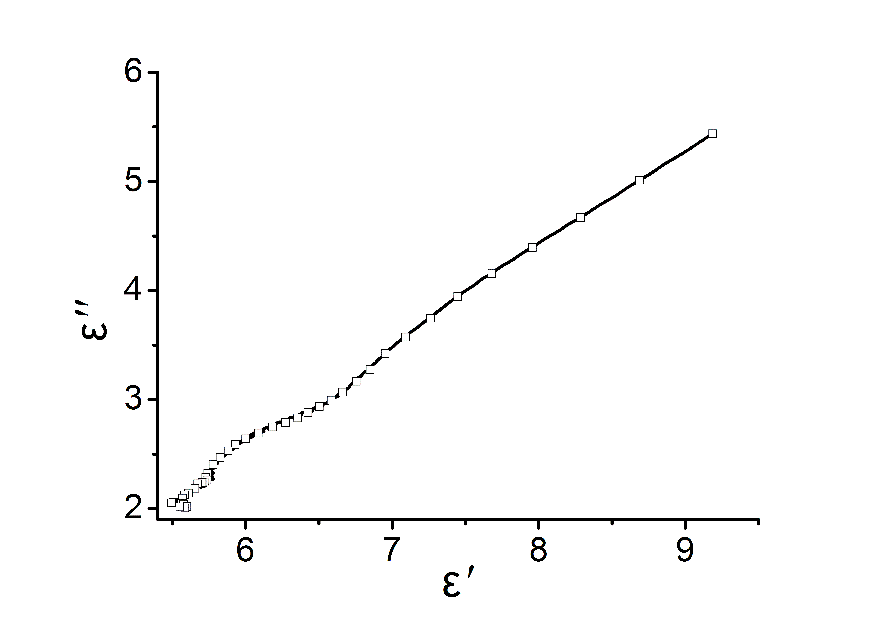


**Figure S2** Cole-Cole plots of CoFe@C.





**Figure S3** The plot of *μ*″(*μ*′)^-2^*f*^-1^ vs. frequency for the CoFe@C.


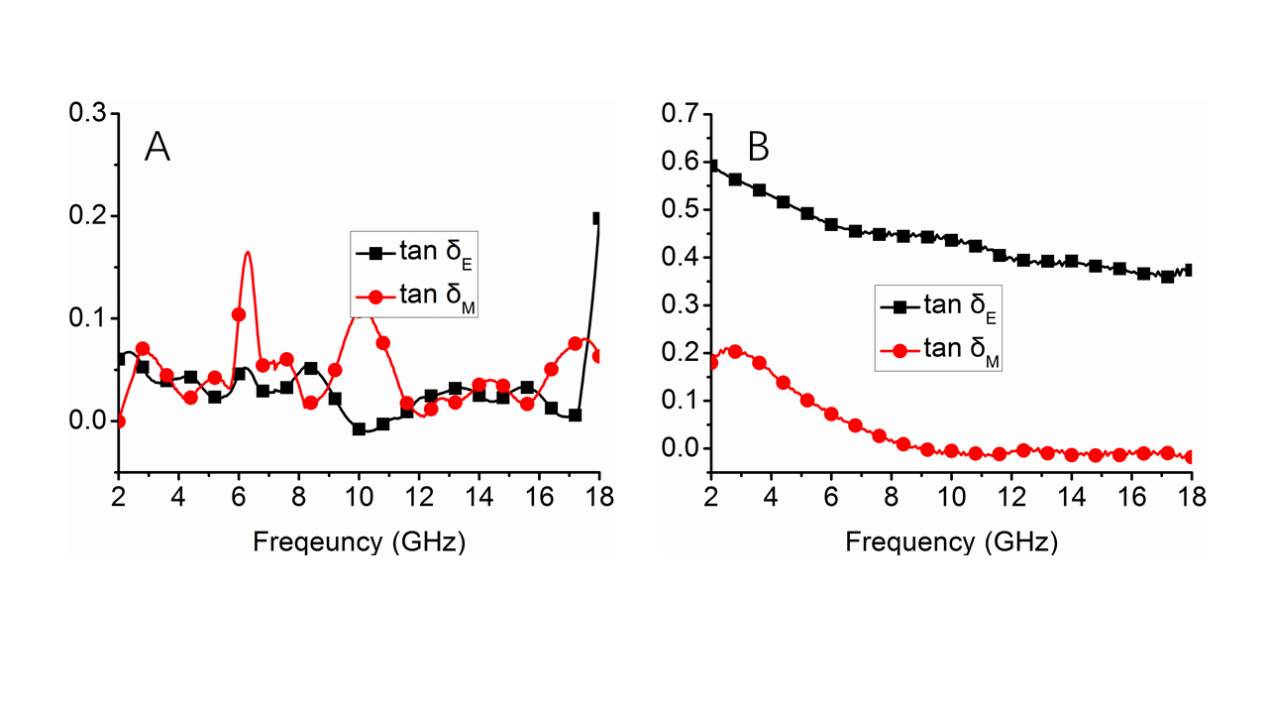


**Figure S4** Frequency dependence of dielectric loss tangents and magnetic loss tangents of the (A) CoFe_2_O_4_ and (B) CoFe@C.
